# Molecular imaging of rheumatoid arthritis: emerging markers, tools, and techniques

**DOI:** 10.1186/ar4542

**Published:** 2014-04-15

**Authors:** Stéphanie Put, René Westhovens, Tony Lahoutte, Patrick Matthys

**Affiliations:** 1 KU Leuven, Laboratory of Immunobiology, Rega Institute, Minderbroedersstraat 10, 3000 Leuven, Belgium; 2 Department of Development and Regeneration; Rheumatology, KU Leuven, Skeletal Biology and Engineering Research Center, University Hospital Leuven, Herestraat 49, 3000 Leuven, Belgium; 3 Department of Nuclear Medicine, UZ Brussel, Laarbeeklaan 101, 1090 Jette, Brussels, Belgium; 4 In Vivo Cellular and Molecular Imaging Center, Vrije Universiteit Brussel, Laarbeeklaan 103, 1090 Jette, Brussels, Belgium

## Abstract

Early diagnosis and effective monitoring of rheumatoid arthritis (RA) are important for a positive outcome. Instant treatment often results in faster reduction of inflammation and, as a consequence, less structural damage. Anatomical imaging techniques have been in use for a long time, facilitating diagnosis and monitoring of RA. However, mere imaging of anatomical structures provides little information on the processes preceding changes in synovial tissue, cartilage, and bone. Molecular imaging might facilitate more effective diagnosis and monitoring in addition to providing new information on the disease pathogenesis. A limiting factor in the development of new molecular imaging techniques is the availability of suitable probes. Here, we review which cells and molecules can be targeted in the RA joint and discuss the advances that have been made in imaging of arthritis with a focus on such molecular targets as folate receptor, F4/80, macrophage mannose receptor, E-selectin, intercellular adhesion molecule-1, phosphatidylserine, and matrix metalloproteinases. In addition, we discuss a new tool that is being introduced in the field, namely the use of nanobodies as tracers. Finally, we describe additional molecules displaying specific features in joint inflammation and propose these as potential new molecular imaging targets, more specifically receptor activator of nuclear factor κB and its ligand, chemokine receptors, vascular cell adhesion molecule-1, α_V_β_3_ integrin, P2X7 receptor, suppression of tumorigenicity 2, dendritic cell-specific transmembrane protein, and osteoclast-stimulatory transmembrane protein.

## Introduction

Anatomical imaging techniques have long been used to diagnose and monitor rheumatoid arthritis (RA). Over the past decade, these techniques have dramatically improved. For example, it is now possible to detect bone erosions within 6 to 8 weeks after arthritis onset. Nevertheless, pure anatomical imaging of even the earliest structural damage misses the preceding molecular, cellular, and physiological changes in the very early stages of RA pathogenesis. Molecular imaging, currently being developed in many domains of medical research and diagnostic practice, offers the possibility to visualize the early functional changes in RA [1]. This non-invasive technique allows early diagnosis, disease monitoring, guidance of treatment strategy, and possibly prediction of the outcome following the selected treatment. For example, patients can be selected for receiving a certain drug on the basis of the presence of the corresponding drug target, as was suggested for treatment of refractory monoarthritis patients with TNF-α antagonists after imaging with ^99m^technetium (Tc)-infliximab [2]. Some RA drugs are relatively expensive; hence, it is important to determine which patients may respond to a proposed therapy and which ones will not. Additionally, patients who are likely to develop a more severe disease can be identified and selected for more intensive treatment or more frequent monitoring. Molecular imaging of joint pathology both in human and in animal models of arthritis will improve our knowledge of the pathogenesis of the disease. In animals, imaging can be performed before and at different time points after the clinical onset of arthritis in the same animal with minimal perturbation of the experiment, and therefore more information can be obtained with fewer animals. Questions such as ‘which are the earliest processes taking place in the pathogenesis of arthritis?’ and ‘which cells are most important in the disease process at which stage?’ might become answered by live-animal imaging with specific probes. In addition, imaging of early-onset inflammation requires sensitive techniques characterized by limited background and non-specific signals.

## Review

### The pathogenesis of arthritis – which cells can we target?

**Figure 1:**
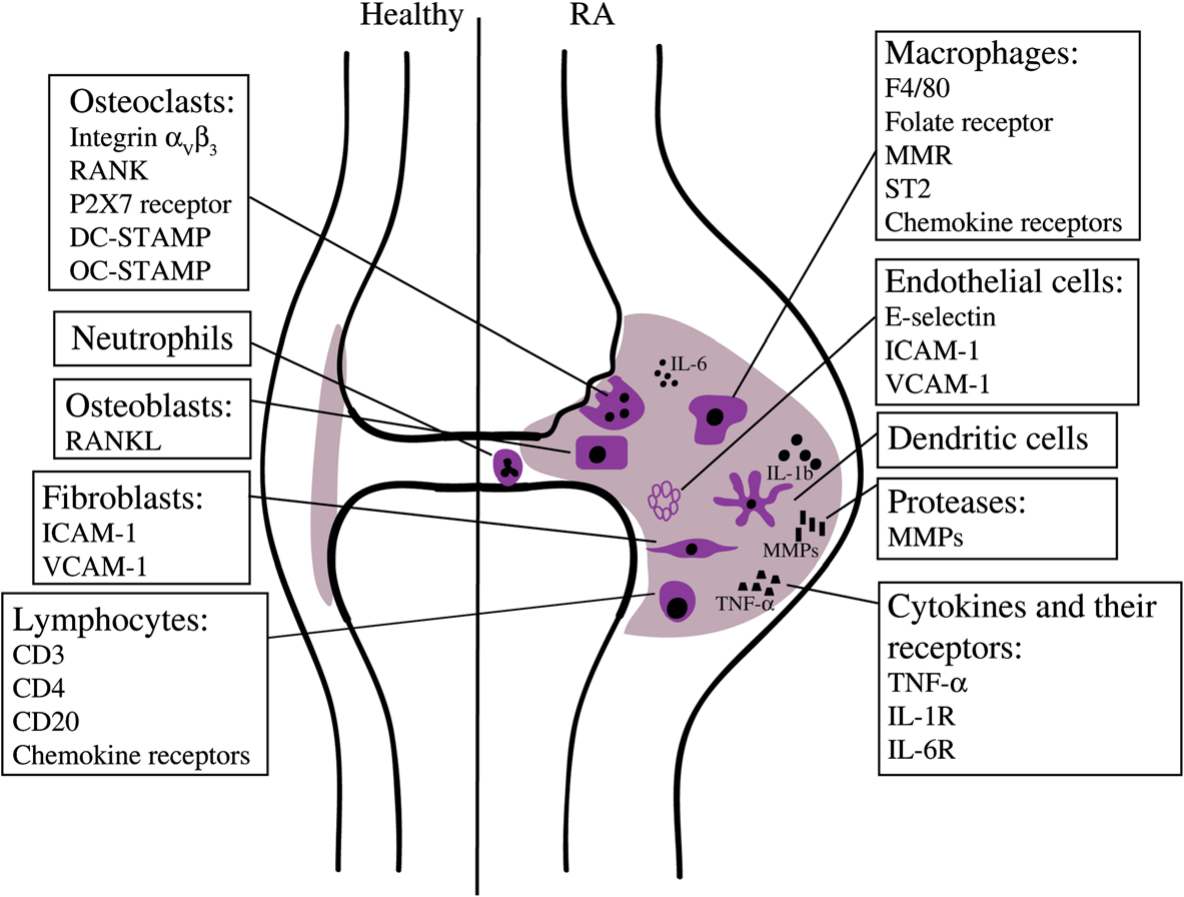
**Schematic overview of specific cells and molecules that can be targeted in the rheumatic joint.** The rheumatoid synovium is characterized by the influx of inflammatory cells and release of cytokines. Surface molecules that are expressed on these cells can be used as markers to target and visualize the different cell types in the inflamed joint. DC-STAMP, dendritic cell-specific transmembrane protein; ICAM-1, intercellular adhesion molecule-1; IL, interleukin; MMP, matrix metalloproteinase; MMR, macrophage mannose receptor; OC-STAMP, osteoclast-stimulatory transmembrane protein; RA, rheumatoid arthritis; RANK, receptor activator of nuclear factor-kappa-B; RANKL, receptor activator of nuclear factor-kappa-B ligand; ST2, suppression of tumorigenicity 2; TNF-α, tumor necrosis factor-alpha; VCAM-1, vascular cell adhesion molecule-1.

RA is a chronic autoimmune disease, affecting approximately 1% of the population worldwide. The disease is characterized by polyarthritis of the diarthrodial joints, primarily the small joints of hands and feet. A hallmark of RA is inflammation of the synovium (synovitis) with influx of mainly macrophages, T cells and B cells [3,4]. The synovial fluid is likewise enriched in immune cells, predominantly neutrophils [5] (Figure 1).

Macrophages are central effectors of synovial inflammation in RA and their abundance and degree of activation are correlated with disease severity [6]. Macrophages act through release of inflammatory factors, phagocytosis, and antigen presentation [4]. In RA, precursors from the monocyte/macrophage lineage are attracted from the blood to the inflamed joint and fuse to become active multinucleated osteoclasts, causing bone destruction.

T cells represent approximately 40% of immune cells in the synovial infiltrate of RA joints and have been implicated in different steps of RA pathology; they promote development of an autoimmune response and production of autoantibodies. Another role of T cells is the production of cytokines and induction of cytokine production by other cells [7]. B cells contribute to RA by the production of autoantibodies, antigen presentation, and T-cell activation [8]. They are indispensable for the development of arthritis as evident from the observation that the depletion of B cells abrogates the development of collagen-induced arthritis (CIA), an animal model for RA [9], and by the effectiveness of the B cell-depleting antibody, rituximab, that binds the CD20 surface molecule on B cells and inhibits the disease [10].

Synovial fibroblasts contribute to RA pathology by releasing matrix-degrading enzymes, including matrix metalloproteinases (MMPs) and cathepsins, which cause cartilage destruction [11]. Osteoblasts differentiate from mesenchymal stem cells and produce bone matrix. At their surface, these cells express receptor activator of nuclear factor-kappa-B ligand (RANKL), which is essential for osteoclast formation [12]. Blood vessel formation is elevated in RA joints and is associated with increased numbers of endothelial progenitor cells. Endothelial cells express cell adhesion molecules that facilitate rolling, binding, and transendothelial migration of leukocytes [13].

## The evolution of arthritis imaging

### Anatomical imaging techniques

Conventional radiography is readily available, inexpensive, and reproducible. It allows the visualization of anatomical changes in established RA, such as erosions, joint space narrowing, and juxta-articular bone loss [14]. Ultrasonography (grey-scale imaging, power Doppler) has been in use for over 30 years. It can be routinely used in the clinic and was found to be more sensitive and accurate than clinical examination or conventional radiography [15]. This technique provides information on bone degradation, synovitis, and inflammation of tendons and entheses. Since ultrasound cannot penetrate bone, osteitis cannot be detected [16]. Practical impediments are the lack of standard protocols for evaluation of RA and, therefore, strict dependence on the physician’s skills. Hence, implementation studies in daily practice are still needed. When magnetic resonance imaging (MRI) was introduced, it soon became evident that this technique outperforms radiography in detecting early bone erosions [17,18]. MRI images are two- and three-dimensional and have a higher contrast resolution, consequently soft tissues can be distinguished more efficiently. With MRI it is possible to detect synovial hyperplasia, bone changes, and cartilage degradation but also signs of RA in the pre-erosive phase [14]. It has now become the gold-standard modality for imaging synovitis in patients with early arthritis. Interestingly, several publications report accurate prediction of radiographic damage by MRI assessment of erosions or inflammation [19,20]. In a study of 42 patients with early RA, baseline MRI erosion scores could predict the development of erosions that became visible by radiography after 1 year. Absence of MRI-detectable erosions predicted the absence of erosions after this time period [21].

### Molecular imaging – state of the art

Structural imaging techniques, though very useful, fail to provide information on the underlying biochemical processes. Therefore, novel imaging modalities using molecular probes such as optical imaging (bioluminescence, fluorescence, and near-infrared, or NIR; 600 to 750 nm) or nuclear imaging (scintigraphy; positron emission tomography, or PET; and single-photon emission computed tomography, or SPECT) are currently being improved for arthritis imaging.

The term optical imaging encompasses techniques, such as bioluminescence and fluorescence, that use light as the primary imaging method. Bioluminescence enables visualization of biological processes *in vivo*. It is a powerful tool for preclinical imaging but less applicable in a clinical setting as it requires administration of foreign enzymes. Fluorescence imaging holds more promise for clinical applications, especially since the development of NIR probes that allow deeper penetration into tissues and less background interference. Nuclear imaging techniques, such as scintigraphy, PET, and SPECT, use markers that are labeled with radioisotopes. Radioactive tracers provide the advantage of deep tissue penetration and low background compared with optical imaging techniques. PET and SPECT produce three-dimensional images and are more sensitive than structural imaging. SPECT is less expensive than PET and uses radiotracers with a longer half-life but has a lower resolution. Additionally, linkage of SPECT tracers requires modifications that might interfere with binding of the marker to the target.

Use of molecular imaging methods might facilitate early diagnosis, disease monitoring, and guidance of treatment strategy, but studies unequivocally demonstrating their value in daily practice are needed. Molecular imaging could be very useful for the selection of patients in phase II clinical trials that evaluate new drugs in a small patient group. The imaging allows measurement of the expression of a given therapeutic target in each individual patient. The level of this expression may be used to select those patients who are most likely to respond to the new therapy. Such an analysis may limit the number of patients who are required in a phase II proof-of-concept trial and may improve the chances of success, but will depend on factors such as the inter-individual variability and the response stratification prediction of the imaging. Imaging is also a powerful tool for animal experimentation; it enables researchers to gather data over a period of time in the same animal. Since the 1990s, molecular imaging has been applied to study arthritis in preclinical imaging of experimental animal models and has evolved from the use of simpler methods, such as autoradiography and planar gamma camera imaging, to more advanced techniques, including fluorescence, PET, and SPECT imaging, that show higher sensitivity and provide more detailed information. The development of the necessary equipment allowing small animal imaging has led to important progress in the field. Fluorescence imaging has greatly advanced as a whole-animal imaging technique and this is due mostly to the development of NIR fluorophores. The background signal in lower wavelengths strongly decreases sensitivity of fluorescence imaging but is significantly less in the NIR range. Nuclear imaging has become more accessible for preclinical research as well; PET and SPECT scanners have been adapted for imaging of small animals.

## Emerging techniques and markers to facilitate molecular imaging of arthritis

### Probes for molecular imaging

**Figure 2:**
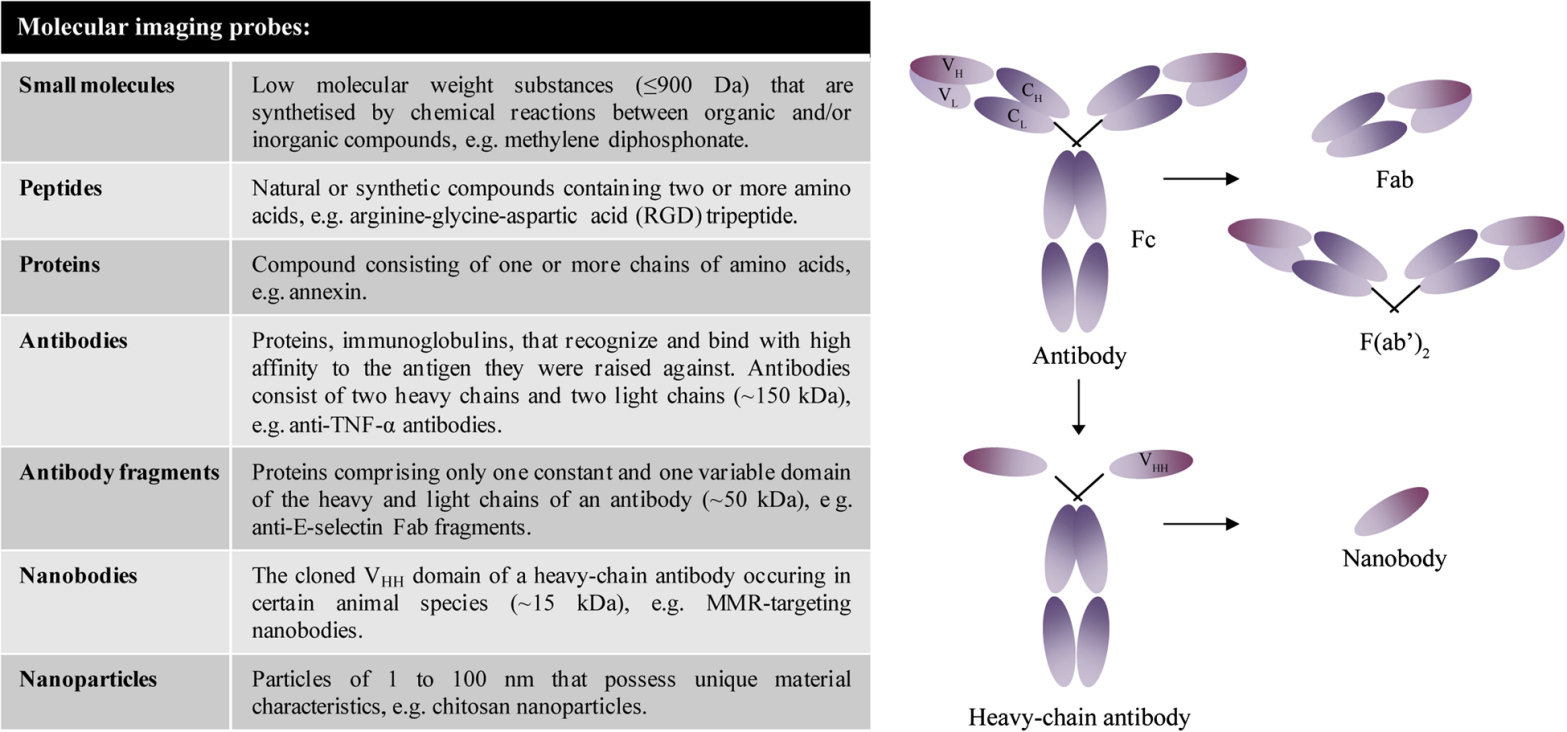
**Probes can be composed of small molecules, peptides, proteins, antibodies, antibody fragments, nanobodies, and nanoparticles.** A schematic overview of a conventional antibody, a heavy-chain antibody, Fab fragments, and a nanobody is given. C_H_, heavy chain constant domain; C_L_, light chain constant domain; Fab, antigen-binding domain; Fc, constant domain; MMR, macrophage mannose receptor; TNF-α, tumor necrosis factor-alpha; V_H_, heavy chain variable domain; V_HH_, heavy chain only antibody V_L_, light chain variable domain.

Molecular imaging probes should hold some key properties (that is, rapid binding with high affinity and specificity for the target, rapid clearance of unbound molecules, high target-to-background ratio, high stability, low immunogenicity and toxicity, and feasibility with respect to production and cost). Probes can be constructed from small molecules, peptides, proteins, antibodies, the antigen-binding region of antibodies (Fab fragments), nanobodies, and nanoparticles (Figure 2). Antibodies are frequently used for specific targeting as they have several advantages over other probes. Generic processes for production of monoclonal antibodies are well established, and monoclonal antibodies are highly specific as they recognize a single molecular epitope. On the downside, antibodies may bind non-specifically via their Fc domains. Antibody administration can trigger undesirable anti-immune responses. To increase target specificity and reduce immunogenicity, Fab fragment probes, comprising only one constant and one variable domain of the heavy and light chains, can be used instead of the complete antibody (Figure 2). An emerging technique in molecular imaging is the use of nanobodies (that is, functional variable fragments of single-chain antibodies that are produced by camelids) (Figure 2). The single-variable domain can be cloned relatively easily from lymphocytes of immunized animals. Nanobodies possess full antigen-binding capacity and are very stable [22]. Their small size (15 kDa) enables them to reach epitopes that are shielded for larger antibodies and additionally allows rapid clearance of unbound tracer from the body. Nanobodies can easily be formatted to meet the needs of several applications [23]. For SPECT imaging, their high intrinsic thermostability and carboxy-terminal hexahistidine tail allow straightforward ^99m^Tc-labeling using tricarbonyl chemistry [24,25]. In addition, nanobodies have been validated as tracers for other imaging modalities (for example, NIR-labeled nanobodies for optical imaging [26] and nanobody-coupled microbubbles for ultrasound [27]). Imaging with labeled nanobodies has proven its value in preclinical models for atherosclerosis and tumors. SPECT imaging with ^99m^Tc-labeled nanobodies targeting vascular cell adhesion molecule-1 (VCAM-1) in apolipoprotein E-deficient mice identified aortic arch atherosclerotic lesions [28]. Nanobodies against the macrophage mannose receptor (MMR) (CD206) were successfully used in SPECT imaging to specifically visualize a subpopulation of tumor-infiltrating macrophages in mammary adenocarcinoma and Lewis lung carcinoma models in mice [29].

Development of new molecular imaging probes for introduction into the clinic is challenging. The regulatory pathway for diagnostics shares features with that for new therapeutics, but the potential revenue from commercialization is lower.

### The use of molecular imaging in the clinic

#### General tracers

**Figure 3:**
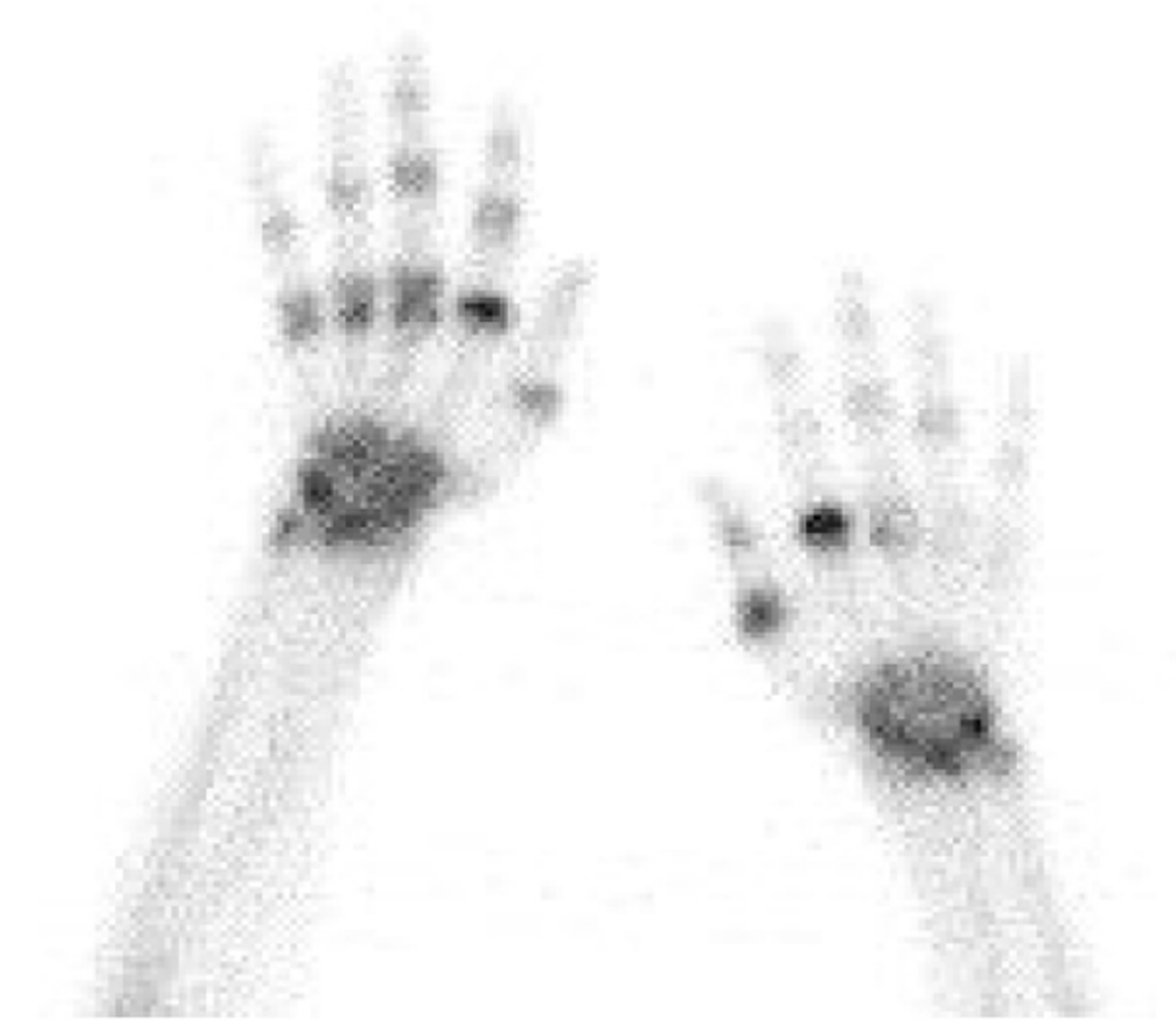
**Bone scintigraphy in a patient with active rheumatoid arthritis.** Imaging was performed at 3 hours after injection of 740 MBq ^99m^technetium-methylene diphosphonate. The image shows increased tracer uptake in the wrists and joints of the fingers. The highest intensity is found in metacarpophalangeal joints.

A frequently used PET tracer is ^18^ F-fluoro-2-deoxy-D-glucose (^18^ F-FDG), which is used to image glucose metabolism. Glucose is normally taken up by cells and phosphorylated by hexokinase. ^18^ F-FDG, on the other hand, cannot be metabolized and therefore accumulates in cells [1]. PET with ^18^ F-FDG is used in experimental models as well as in the clinic to study inflammation of joints by detecting its accumulation in activated macrophages, neutrophils, and proliferating fibroblasts [1,30]. The technique has been successfully used to image RA-related inflammation and even to predict the response to therapy of patients with RA. A correlation was noted between PET activity 2 weeks after initiation of infliximab treatment and the disease activity score 28 after 14 and 22 weeks of treatment [31]. Increased uptake of ^18^ F-FDG in the joints is not specific for RA, as this also occurs in infectious and degenerative forms of osteoarthritis [32]. Radiolabeled diphosphonates do not locate primarily to inflammatory sites, but detect alterations in bone metabolism, especially increased pathological osteoblast activity [33,34]. SPECT imaging with ^99m^technetium-methylene diphosphonate (^99m^Tc-MDP) is clinically approved for the assessment of bone damage in RA and is used to monitor patients with active arthritis [33] (Figure 3). This tracer has proven to be useful for arthritis imaging in the past but provides limited insight into the disease process. Furthermore, uptake of ^99m^Tc-MDP occurs in all joints, making discrimination with mild arthritis difficult and precluding differentiation between active synovitis and inflammation secondary to joint destruction in chronically affected joints. Other general probes (discussed in Table 1) have yielded positive results in trials with patients with RA, but many have not been further developed for this application, mostly as a result of the limited information they provide in addition to clinical assessment in comparison with more specific probes. Consequently, additional imaging probes are being evaluated for assessment of synovitis in patients.

**Table 1:** Available imaging agents for rheumatoid or experimental arthritis

**Tracer**	**Target**	**Developmental phase**	**Reference**
**General tracers**			
^18^ F-fluoro-2-deoxy-D-glucose	Glucose metabolism, inflammation	Clinical use for detection of inflammation and cancer	[1,35]
^99m^Tc-diphosphonates	Alterations in bone metabolism, osteoblastic activity	Clinical use for bone scanning	[33,34]
^11^C-choline	Cell membrane synthesis, inflammation	Clinical use for detection of prostate cancer	[36,37]
		Trial in 10 patients with inflammatory joint disease	
^67^Ga-citrate	Circulating blood plasma proteins and leukocytes, inflammation	Clinical use for detection of inflammation	[14]
^99m^Tc-polyclonal human immunoglobulin G	Increase in vascular permeability, hyperemia, inflammation	Multiple trials in patients with rheumatic disorders	[38,39]
^99m^Tc-/^111^In-labeled leukocytes	Influx of leukocytes into inflamed tissue	Clinical use for infectious and inflammatory disorders	[40]
		Multiple trials in patients with RA	
^99m^Tc-RP128	Leukocytes (binds to receptors on neutrophils and mononuclear phagocytes)	Trial with 10 patients with RA	[41]
**Radiolabeled biologicals**			
^99m^Tc-anti-CD4 mAb	T cells	Case study in 1 patient with RA, trial in 6 patients with RA	[42-45]
^99m^Tc-anti-CD3 mAb	T cells	Trials in 7 patients with RA, 2 psoriatic arthritis patients, and 38 patients with RA	[46-48]
^123^I-IL-1Ra	Inflammation	Trial in 4 patients with active RA	[49]
^99m^Tc-anti-TNF-α	Inflammation	Multiple trials with patients with RA	[2,50-52]
		Phase 3 study (NCT01590966)	
^99m^Tc-/^124^I-anti-CD20 mAb	B cells	Trials in 6 patients with RA and 20 patients with chronic inflammatory autoimmune disease	[53,54]
**Specific molecular markers**			
^99m^Tc-acetylated poly-(1,3)-D-galactoside	Mononuclear phagocyte trafficking (binds CD14 and CD11b)	Trials for tumoral, inflammatory and infectious diseases	[55]
		Preclinical, rabbit antigen-induced arthritis	
^99m^Tc-/^111^In-octreotide	Endothelium activation and macrophage recruitment (binds to somatostatin receptor)	Clinical use for detection of tumors	[56]
		Trial in 14 patients with RA	
^11^C-(R)-PK11195	Monocytes and macrophages (binds to peripheral benzodiazepine receptors)	Trials in 11 patients with RA, 6 patients with RA, and 29 patients with arthralgia	[57,58]
^111^In-E-selectin-binding peptide	Activated vascular endothelium	Preclinical, rat adjuvant arthritis	[59]
^99m^Tc-anti-E-selectin Fab/(Fab′)_2_ fragment	Activated vascular endothelium	Trial in 26 patients with RA	[60]
NIR-anti-E-selectin Ab	Activated vascular endothelium	Preclinical, CIA, and TNF-α-induced paw edema	[61]
^99m^Tc-annexin V	Apoptosis (binds to phosphatidylserine)	Multiple trials in cancer patients	[62]
		Preclinical, CIA	
Cy5.5-anti-F4/80	Macrophages	Preclinical, AIA	[63]
^99m^Tc-anti-IL-6R	Inflammation	Preclinical, murine arthritis model	[64]
^99m^Tc-folic acid (EC20)	Activated macrophages, folate receptor	Phase 2 study in patients with autoimmune disease (NCT00588393)	[65-67]
NIR2-folate	Activated macrophages, folate receptor	Mouse arthritis models	[68]
^18^ F-PEG-folate	Activated macrophages, folate receptor	Preclinical, methylated bovine serum albumin-induced arthritis	[69]
^64^Cu-/^18^ F-galacto-arginine-glycine-aspartic acid	Activated macrophages, osteoclasts, endothelial cells	Clinical use, tumor angiogenesis	[70]
		Preclinical, osteopetrosis, and osteoporosis mouse models	
NIR-matrix metalloproteinase-specific probe	Sites of matrix degradation and inflammation	Preclinical, CIA, rat OA	[71]
^99m^Tc-anti-macrophage mannose receptor	Subset of macrophages	Preclinical, CIA	[72]

^99m^Tc, ^99m^technetium; Ab, antibody; AIA, antigen-induced arthritis; CIA, collagen-induced arthritis; IL, interleukin; mAb, monoclonal antibody; NIR, near-infrared; OA, osteoarthritis; RA, rheumatoid arthritis; TNF-α, tumor necrosis factor-alpha.

#### Radiolabeled biologicals

Several biologicals approved for the treatment of RA have been radiolabeled and evaluated for imaging, and this provides a number of advantages. These drugs are already being developed commercially, and their safety has been assessed. Radiolabeled therapeutics might provide an earlier, more specific diagnosis and facilitate monitoring of treatment efficacy. Importantly, confirmation of the presence of the target in the patient before treatment initiation provides the possibility of personalized therapy, which can significantly reduce treatment costs. In this context, anti-CD3 [46-48], anti-CD4 [42-45], anti-CD20 [53,54], and anti-TNF-α (infliximab [2,50] and adalimumab [51,52]) have been evaluated for imaging of RA, and most perform relatively well. An exception is IL-1 receptor antagonist (anakinra), which failed to show any specific localization in synovia of RA patients as compared with healthy synovia [49]. As evident from clinical trials, uptake of radiolabeled monoclonal anti-CD3 antibodies (OKT-3), which target all T cells, was detectable in inflamed joints of patients with RA and levels correlated with inflammation scores from physical examination [46-48]. Anti-CD4 antibodies (MAX.16H5) target a more specific subset of T lymphocytes. These antibodies were also evaluated for visualization of inflammatory foci in patients with RA [42-45]. Early studies on the specificity of the antibody signal yielded conflicting results [73], but this specificity was later confirmed in a study using Fab fragments. A higher accumulation of anti-CD4 Fab′ fragments compared with control Fab′ fragments was clearly shown in adjuvant-induced arthritis [74]. Rituximab, a chimeric monoclonal anti-CD20 antibody, is licensed for treatment of RA and has demonstrated good efficacy in a subset of patients [75]. ^99m^Tc-rituximab was shown to localize in inflamed joints of patients with RA [53,54]. Here, an interesting intra-articular and inter-individual variability was exposed, providing an explanation for the failure of anti-CD20 therapy in certain patients [54]. Finally, with respect to anti-TNF-α, imaging of patients with active RA by using ^99m^Tc-anti-TNF-α showed a high correlation with inflammation detected by MRI and proved to be more sensitive than clinical examination [52]. In addition, Conti and colleagues [2] reported that imaging with anti-TNF-α in patients with active arthritis just before treatment with infliximab could predict the efficacy of the anti-TNF-α therapy.

As evident from Table 1, these radiopharmaceuticals have been evaluated in trials dating back several years; for some of them, no real implementation in imaging has been reported in recent years. Further development of radiolabeled drugs for imaging purposes is subjected to important limitations: (a) the safety issues of murine and chimeric monoclonal antibodies; problems with immunogenicity are now being handled by humanization of the antibodies. (b) The safety profile of the pharmaceutical. Administration of anti-CD3 antibodies, for example, may cause serious adverse effects in some patients, such as the cytokine release syndrome. (c) The inefficacy of certain drugs in patients with RA. As mentioned, anti-CD4 antibody was demonstrated to have potential for location of inflammatory regions, but the lack of therapeutic results in RA has halted further development for imaging. (d) The high costs associated with development and production of these therapies.

In our opinion, radiolabeled anti-CD20 and anti-TNF-α antibodies show the most potential for clinical use. Their effectiveness as treatment for RA has been established, and the first results on identification of patients who will respond to therapy look promising. Further implementation studies demonstrating their value in selecting patients for specific therapies are needed. Currently, conflicting results are found in the literature - coming mostly from registries - about switching to another mode of action or to another TNF blocker in patients failing their first anti-TNF treatment [76,77]. In the future, the use of radiolabeled antibodies in appropriate randomized studies could help to produce more solid data that might give guidance to clinicians.

### Specific tracers for imaging of arthritis in preclinical models

Molecular imaging in animal models is necessary for the development of new tracers but can also be used as an additional objective parameter to assess arthritis in an experimental context, thereby providing detailed information on the disease process. Tracers have evolved to target molecules more specifically than ^18^ F-FDG or ^99m^Tc-MDP, which visualize general processes (glucose metabolism and bone turnover), and we can see an evolution in the use of more sophisticated techniques for preclinical research. An example of the technical evolution can be seen in the development of tracers that target the folate receptor, a 38-kDa glycosyl-phosphatidylinositol-anchored protein that binds folic acid [65]. The folate receptor is expressed at very low levels in most tissues in homeostatic conditions, except for the kidneys and placenta. Under these circumstances, folic acid is taken up by carriers [78]. Expression of the folate receptor in pathogenic conditions seems to be restricted to several cancer cells and activated macrophages. Interestingly, high-level expression of this receptor was found in activated synovial macrophages from patients with RA [79], a feature that has been exploited in imaging. In 2002, Turk and colleagues [66] published a study in which the folate receptor was targeted in rats with adjuvant-induced arthritis by using ^99m^Tc-labeled folic acid. Gamma scintigraphy was applied to produce images of the inflamed paws, and uptake could be detected in arthritic joints [66]. However, the resolution of this technique is very low. Chen and colleagues [68] developed a fluorescence-labeled folate probe (NIR2-folate) that allowed improved resolution in two mouse models of arthritis and the possibility of detecting arthritis at an early time point. So far, however, fluorescent imaging is being hindered by limited tissue penetration and is not yet optimized for use in patients with RA. Recently, an improved PET tracer (^18^ F-polyethylene glycol-folate) targeting the folate receptor was shown to hold promise for imaging RA in patients, as it was successfully used in an antigen-induced arthritis model [69]. A folate receptor-targeting agent, ^99m^Tc-EC20 (FolateScan), has been produced and evaluated in the clinic for the assessment of inflammation in joints of patients with RA or other diseases [67,80]. In a study including 40 RA patients with active and inactive disease, joint involvement was assessed by screening with FolateScan. The number of actively involved joints identified by FolateScan correlated with erythrocyte sedimentation rates and C-reactive protein levels. Larger numbers of actively involved joints were detected with FolateScan than were identified by clinical examination. It was concluded that imaging with FolateScan may be a more sensitive method than physical examination for assessing disease activity [67]. Upregulation of the folate receptor in activated macrophages is also being exploited for targeting of folate-linked drugs to these macrophages in RA [81,82]. Imaging with FolateScan might help to predict the success rate of this technique.

**Figure 4:**
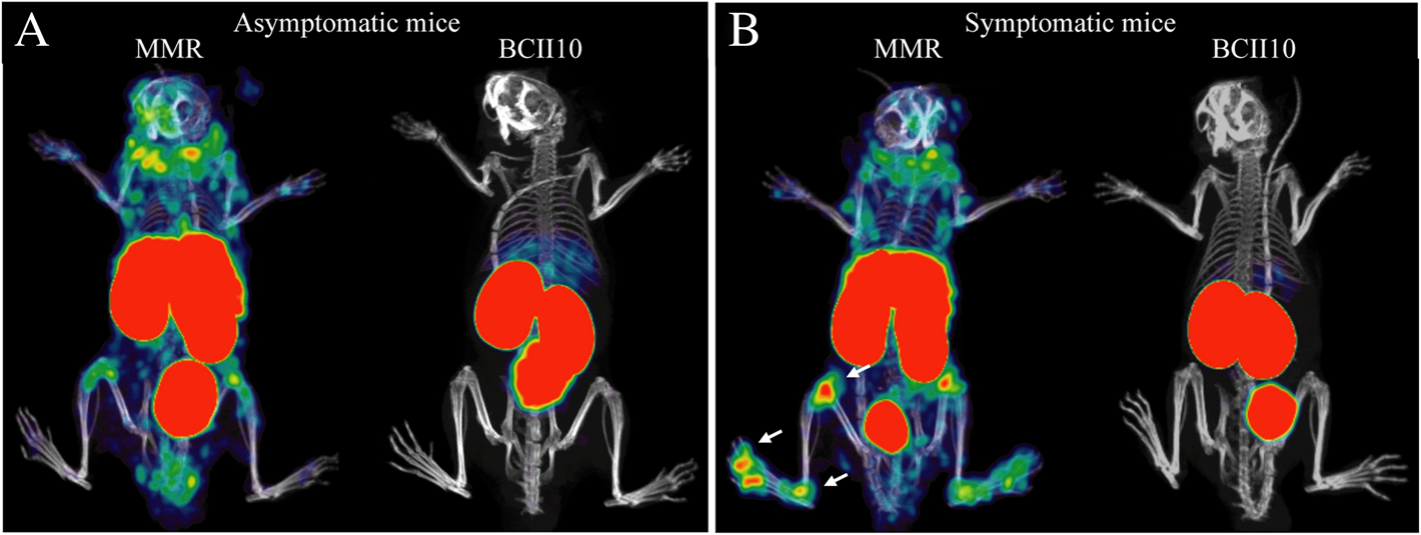
***In vivo *imaging with macrophage mannose receptor (MMR)-specific nanobodies visualizes MMR expression in joints of mice with collagen-induced arthritis.** Single-photon emission computed tomography and micro-computed tomography imaging was performed at 3 hours after injection of ^99m^technetium-labeled MMR-targeting nanobodies in mice without clinical symptoms of arthritis **(A)** (asymptomatic mice) or mice with arthritic joints **(B)** (symptomatic mice). Nanobodies against the β-lactamase BCII enzyme of *Bacillus cereus* (BCII10) were used as a non-targeting control. MMR staining is apparent in knees, ankles, and metatarsal joints of symptomatic mice (arrows) in addition to the signal in lymph nodes, liver, and spleen that is also detectable in asymptomatic mice. This image was originally published in the *Journal of Nuclear Medicine*[72]. © by the Society of Nuclear Medicine and Molecular Imaging, Inc.

F4/80, a member of the epidermal growth factor transmembrane 7 family, is expressed on a variety of macrophage subsets. Macrophages that accumulate in inflamed joints express F4/80. In a mouse model, imaging with NIR-labeled antibodies targeting F4/80 visualized macrophage accumulation in arthritic joints, with some background in healthy paws [63]. The MMR is a 175-kDa C-type lectin expressed predominantly by mature macrophages and certain endothelial and dendritic cells. It is detected in spleen, liver, and lymph nodes and its primary functions are endocytic clearance of certain glycoproteins and phagocytosis of unopsonized microorganisms [83]. Our research group recently reported the successful use of radiolabeled nanobodies targeting MMR for *in vivo* SPECT/CT imaging of mice with CIA (Figure 4) [72]. We were able to visualize CD11b^+^F4/80^+^ macrophages in the inflamed joints of these mice, thereby providing a means to quantify the inflammation in an objective manner and obtaining more knowledge on the pathogenesis of arthritis, since MMR had previously not been shown to be expressed in the rheumatic synovium [72].

Vascular endothelium, activated in inflammatory processes, transiently expresses the surface glycoprotein E-selectin in response to cytokines such as IL-1β and TNF-α. Selectins facilitate tethering and rolling of leukocytes on endothelium. These mechanisms commence early in the pathogenesis of arthritis. Fluorescently labeled anti-E-selectin antibodies were successfully used for imaging in a mouse model for RA, enabling detection of subclinical manifestations, monitoring effects of therapy, and quantification of disease [61]. In patients with RA, ^99m^Tc-anti-E-selectin-Fab appeared to be suitable for scintigraphic imaging of synovitis and showed a higher specificity than ^99m^Tc- hydroxymethylene diphosphonate bone imaging [60].

Intercellular adhesion molecule-1 (ICAM-1) is another potential target for imaging of arthritis as it was demonstrated to be expressed on synovial endothelial cells and mice deficient in ICAM-1 showed a reduction in arthritis incidence and severity [84]. In CIA, antibodies targeting ICAM-1 were conjugated to gadolinium diethylenetriamine pentaacetic acid and were able to detect early inflammatory symptoms before the onset of the chronic destructive phase [85].

In arthritic joints, increased apoptotic cells are detected in the synovial membrane [86]. These cells can be imaged by the use of radiolabeled annexin V that binds to phosphatidylserine, which is associated with the inner leaflet of the plasma membrane. It was found that ^99m^Tc-annexin could visualize arthritic joints before the onset of bone destruction [62].

Inflammatory cytokines, such as IL-1β and TNF-α, stimulate the production of MMPs that degrade the extracellular matrix. Levels of MMPs increase in the serum and the synovial fluid of patients with RA. MMP-1, −2, −3, −9, and −13 appear to be most important in RA. Recently, Ryu and colleagues [71] demonstrated the use of an MMP-3-specific polymeric probe for visualization of arthritis by NIR fluorescence imaging. The probe was developed by the conjugation of a NIR dye, an MMP substrate peptide, and a quencher to chitosan nanoparticles. Imaging with this probe allowed early diagnosis of arthritis in mice with CIA. More specifically, at 2 weeks after immunization, before any signs of structural or anatomical changes, a signal from the MMP-3 probe could be detected [71].

Development of these molecular markers is still in its infancy; many have only recently been evaluated for imaging of arthritis in preclinical models. Some show potential for further development, such as MMP-specific probes, which are being commercialized and are entering research labs. Others are less promising for the future and this is due mostly to lower specificity. The targeted cell type might not be exclusively expressed in the inflamed joint or the probe might bind to irrelevant targets, resulting in non-specific signals and low target-to-background ratios.

### Potential new targets to be considered for *in vivo* imaging of arthritis

Molecules in the following section were chosen for discussion because they have been shown, or suggested, to be involved in the pathogenesis of arthritis through functional preclinical studies and expression studies in animal models or patients.

#### RANK and RANKL

RANKL is a type I-membrane protein of the TNF receptor superfamily, expressed on osteoblasts and T cells. RANKL knockout mice exhibit severe osteopetrosis because of a complete absence of osteoclasts. Furthermore, the importance of RANK-RANKL signaling for bone destruction in arthritis has been demonstrated in several studies [87,88]. Inhibition of RANKL by denosumab, a human monoclonal antibody, has been effective for the treatment of RA-associated bone loss [89] and bone metastasis in cancer [90]. The receptor for RANKL, RANK, can be detected on pre-osteoclasts in the blood as well as on mature osteoclasts [91]. Importantly, RANK has already proven to be a potential marker for the detection of bone metastasis [92-94]. RANK and RANKL can thus be considered valuable molecular targets for the treatment of bone loss and have great value as markers for RA-associated bone pathology.

#### Chemokine receptors

Chemokines and their receptors are involved in the recruitment of leukocytes to the site of inflammation; they are key molecules in the pathogenesis of RA and present possible targets for imaging. CCR1 and CCR5 are the ones most implicated in RA. CCR1 is abundantly expressed by macrophages in the inflamed synovium and peripheral blood monocytes of patients with RA, suggesting an important role in recruitment of leukocytes from the circulation. A trial involving 160 patients with RA showed evidence of a beneficial effect of treatment with the small-molecule CCR1 antagonist CCX354-C [95]. CCR5 is highly expressed in the rheumatoid synovium, particularly by T helper (Th)1 lymphocytes [96]. Antagonists of CCR5 have proven to be capable of inhibiting the development of collagen- and adjuvant-induced arthritis [97,98].

#### Surface molecules involved in cell adhesion and cell signaling

VCAM-1 is thought to be responsible for recruitment and retention of leukocytes in the inflamed synovium. It is found on fibroblast-like synoviocytes in the synovial lining layer and is upregulated upon stimulation with various cytokines [99]. As mentioned, nanobodies targeting VCAM-1 have proven their worth in imaging of atherosclerotic lesions. The expression of VCAM-1 in arthritis suggests that these nanobodies might also be useful in imaging of arthritic joints.

Integrins, a large family of heterodimeric transmembrane glycoproteins, mediate cell-cell and cell-matrix interactions. Their expression is upregulated in the pro-inflammatory environment of the rheumatoid synovium and leads to production of matrix-degrading enzymes and cytokines [100]. Vitaxin, a humanized monoclonal antibody that blocks the interactions of α_v_β_3_ with its ligands, was tested as treatment for RA in clinical trials [101]. Mature and active osteoclasts express α_v_β_3_; consequently, inhibition or deficiency of the β_3_ integrin was shown to cause impaired differentiation and function of osteoclasts [102]. Imaging studies were performed with the tripeptide Arg-Gly-Asp (RGD), which binds with high affinity to α_v_β_3_. PET imaging with ^64^Cu-labeled RGD allowed detection of changes in osteoclast numbers in mouse models for osteopetrosis or osteoporosis [70], suggesting that ^64^Cu-RGD may be suitable for imaging of osteoclast changes in RA.

Another cell surface molecule implicated in the pathogenesis of RA is the purinoreceptor P2X7, which was shown to be expressed by synoviocytes from RA joints. P2X7 is expressed by various cells, including osteoblasts and osteoclasts, and has been implicated in the formation of multinuclear cells. Triggering of this receptor results in enhanced IL-6 secretion and its absence was shown to result in a loss of ATP-dependent leukocyte function, including IL-1β production and L-selectin shedding [103]. In animal models of arthritis, deficiency of the P2X7 receptor is associated with lower incidence and severity of arthritis [104].

Suppression of tumorigenicity 2 (ST2) is a member of the Toll-like/IL-1 receptor superfamily that participates in inflammatory processes, such as production of Th2 cytokines. The ST2/IL-33 pathway has been implicated in RA pathogenesis, and treatment of CIA with ST2-Fc fusion protein ameliorated the disease and downregulated production of IL-6, IL-12, and TNF-α [105].

Dendritic cell-specific transmembrane protein (DC-STAMP) and osteoclast-stimulatory transmembrane protein (OC-STAMP) are highly expressed by osteoclasts and essential for the fusion of macrophages to multinuclear cells and bone degradation [106]. DC-STAMP was suggested to be a marker for circulating osteoclast precursors in inflammatory arthritis as it was found to be increased on peripheral blood mononuclear cells of patients with psoriatic arthritis [107]. DC-STAMP and OC-STAMP could be valuable for therapy development and imaging of osteoclasts in arthritis.

### Challenges for development of new molecular probes

High selectivity of molecular imaging probes is mandatory and represents the most important aspect to consider when developing new candidates for imaging. A second challenge to consider is resolution; theoretically, molecular imaging could also help in showing specific localizations of inflammation, distinguishing enthesitis from arthritis in spondyloarthritis versus RA, but this would require techniques with better resolution. To qualify the cost-effectiveness of a new marker, the molecular imaging method should have an impact on patient management. As stated above, molecular imaging methods might be of help in designing and evaluating proof-of-concept phase I and II trials assessing specific targets. The use of these tools, however, will become established only if they facilitate daily treatment practice in making an appropriate choice for the best treatment option or are able to guide the physician in tapering expensive treatment if a specific state of prolonged remission has been reached. Ultimately, as stated in a recent editorial by Smolen and Aletaha [108], better markers will be needed to make a transition toward real personalized medicine.

## Conclusions

The field of molecular imaging has grown substantially in the past two decades; tools and techniques have evolved, and new molecular markers are being identified. Molecular imaging will be of help in a preclinical context by offering a reliable and subjective manner to assess the disease severity and by providing more detailed knowledge of the disease process. Some of the markers will enter the clinic to facilitate diagnosis, monitoring of disease progression, and determination of treatment strategy in a subset of patients. Finally, an important role for molecular imaging may be situated in the assessment of efficacy of new drugs and in the design and evaluation of clinical trials.

## Abbreviations

18 F-FDG: ^18^fluoro-2-deoxy-D-glucose; 99mTc-MDP: ^99m^technetium-methylene diphosphonate; CIA: Collagen-induced arthritis; CT: Computed tomography; DC-STAMP: Dendritic cell-specific transmembrane protein; ICAM-1: Intercellular adhesion molecule-1; IL: Interleukin; MMP: Matrix metalloproteinase; MMR: Macrophage mannose receptor; MRI: Magnetic resonance imaging; NIR: Near-infrared; OC-STAMP: Osteoclast-stimulatory transmembrane protein; PET: Positron emission tomography; RA: Rheumatoid arthritis; RANK: Receptor activator of nuclear factor-kappa-B; RANKL: Receptor activator of nuclear factor-kappa-B ligand; RGD: Arg-Gly-Asp; SPECT: Single-photon emission computed tomography; ST2: Suppression of tumorigenicity 2; Tc: Technetium; Th: T helper; TNF-α: Tumor necrosis factor-alpha; VCAM-1: Vascular cell adhesion molecule-1.

## Competing interests

The authors declare that they have no competing interests.
